# Comparative Plastid Genomics of Non-Photosynthetic Chrysophytes: Genome Reduction and Compaction

**DOI:** 10.3389/fpls.2020.572703

**Published:** 2020-09-10

**Authors:** Jong Im Kim, Minseok Jeong, John M. Archibald, Woongghi Shin

**Affiliations:** ^1^ Department of Biology, Chungnam National University, Daejeon, South Korea; ^2^ Department of Biochemistry and Molecular Biology, Dalhousie University, Halifax, NS, Canada

**Keywords:** genome reduction, leucoplast, non-photosynthesis, chrysophytes, plastid genome

## Abstract

*Spumella*-like heterotrophic chrysophytes are important eukaryotic microorganisms that feed on bacteria in aquatic and soil environments. They are characterized by their lack of pigmentation, naked cell surface, and extremely small size. Although *Spumella*-like chrysophytes have lost their photosynthetic ability, they still possess a leucoplast and retain a plastid genome. We have sequenced the plastid genomes of three non-photosynthetic chrysophytes, *Spumella* sp. Baeckdong012018B8, *Pedospumella* sp. Jangsampo120217C5 and *Poteriospumella lacustris* Yongseonkyo072317C3, and compared them to the previously sequenced plastid genome of “*Spumella*” sp. NIES-1846 and photosynthetic chrysophytes. We found the plastid genomes of *Spumella*-like flagellates to be generally conserved with respect to genome structure and housekeeping gene content. We nevertheless also observed lineage-specific gene rearrangements and duplication of partial gene fragments at the boundary of the inverted repeat and single copy regions. Most gene losses correspond to genes for proteins involved in photosynthesis and carbon fixation, except in the case of *pet*F. The newly sequenced plastid genomes range from ~55.7 kbp to ~62.9 kbp in size and share a core set of 45 protein-coding genes, 3 rRNAs, and 32 to 34 tRNAs. Our results provide insight into the evolutionary history of organelle genomes *via* genome reduction and gene loss related to loss of photosynthesis in chrysophyte evolution.

## Introduction

Chrysophytes are a large algal group with diverse morphologies and various nutritional modes, including phototrophy, mixotrophy, and heterotrophy. Among them, mixotrophs and heterotrophs are ecologically important eukaryotes that feed on bacteria and other eukaryotes inhabiting freshwater, brackish, and marine environments. The phototrophic chrysophytes have golden-brown plastids with chlorophylls *a* and *c*. The chrysophyte plastid is derived from a red alga through secondary (i.e., eukaryote-eukaryote) endosymbiosis (Kim and Archibald, 2009). As stramenopiles, chrysophytes are closely related to the Synchromophyceae and Eustigmatophyceae. They are also phylogenetically grouped together with 16 classes of plastid-containing stramenopiles, including brown algae and diatoms (Han et al., 2019; Kim et al., 2019).

Non-photosynthetic chrysophytes appear to have evolved several times independently within the chrysophytes (Grossmann et al., 2016). *Spumella*-like species, which are non-photosynthetic nanoflagellates, are characterized by colorless cells with naked cell surfaces, a non-colonial lifestyle, heterokont flagella, and endogenous silicified stomatocysts. Recent taxonomic studies based on nuclear small subunit (SSU) rDNA sequence data suggest that *Spumella*-like flagellates are not monophyletic and have lost the ability to photosynthesize many times in different lineages, resulting in seven new *Spumella*-like genera (Boenigk and Stadler, 2004; Findenig et al., 2010; Grossmann et al., 2016). Molecular phylogenies clearly show that these independently evolved colorless chrysophyte species are related to photosynthetic lineages, including phototrophic and mixotrophic species, within chrysophytes (Grossmann et al., 2016; Andersen et al., 2017; Dorrell et al., 2019). Interestingly, non-pigmented, vestigial plastids (leucoplasts) have been reported in *Poteriospumella lacustris*, *Cornospumella fuschlensis*, *Spumella vulgaris*, and *Pedospumella encystans*, suggesting that phagotrophic *Spumella*-like flagellates are derived from ancestral phototrophic or mixotrophic species with photosynthetic ability (Grossmann et al., 2016).

The loss of photosynthesis in autotrophic organisms containing primarily and secondarily derived plastids has occurred multiple times during the course of eukaryotic evolution (Kim and Archibald, 2009; Hadariová et al., 2018; Maciszewski and Karnkowska, 2019; Sibbald and Archibald, 2020). Although the loss of most or all photosynthesis-related genes leads to a reduction in plastid genome size and complexity, most non-photosynthetic taxa retain a plastid genome. Such examples include the green algal pathogens *Helicosporidium* sp. (de Koning and Keeling, 2004) and *Prototheca* spp. (Knauf and Hachtel, 2002; Borza et al., 2005; Suzuki et al., 2018), the parasitic plant *Epifagus virginiana* (de Pamphilis and Palmer, 1990), members of the free-living green algal genus *Polytoma* (Nedelcu, 2001; Figueroa-Martinez et al., 2017), and the euglenoid alga *Euglena longa* (Gockel and Hachtel, 2000). Additionally, genome-containing non-photosynthetic plastids of red-algal secondary endosymbiotic origin are diverse in nature, and can be found in the malaria parasite *Plasmodium falciparum* (Wilson et al., 1996; Waller and McFadden, 2005), the cryptophyte *Cryptomonas paramecium* (Donaher et al., 2009; Tanifuji et al., 2020), the colorless diatom *Nitzschia* species. (Kamikawa et al., 2018), and the chrysophyte “*Spumella*” sp. NIES-1846 (Dorrell et al., 2019).

Many comparative genomic and phylogenomic studies have used plastid genome data to shed light on the evolution of photosynthetic stramenopiles (Ruck et al., 2014; Ševčíková et al., 2015; Han et al., 2019; Kim et al., 2019). In non-photosynthetic species of chrysophytes, the ultrastructures of remnant plastids have been reported (Grossmann et al., 2016), and a recent genome and transcriptome-based phylogenomic study suggested parallel reductive evolution of non-photosynthetic chrysophytes, including the genus *Paraphysomonas*, which appears to have undergone complete loss of the plastid genome (Dorrell et al., 2019). But there are still many unanswered questions about the loss of photosynthesis in the chrysophytes, questions that can be addressed by investigating the structure and coding capacity of plastid genomes in diverse chrysophyte species.

To that end, we sequenced three plastid genomes from the following representative non-photosynthetic chrysophyte algal genera: *Spumella, Pedospumella*, and *Poteriospumella*. We carried out a detailed comparative analysis of their plastid genome structures and gene contents relative to each other and to published photosynthetic chrysophyte plastid genome sequences for *Ochromonas* sp. CCMP1393, synuralean algae, and the non-photosynthetic “*Spumella*” sp. NIES-1846 (Ševčíková et al., 2015; Dorrell et al., 2019; Kim et al., 2019). Our results contribute to the growing body of knowledge relating to how gene content and genome structure changes in response to the loss of photosynthesis in chrysophytes and other algae.

## Materials and Methods

### Cultures, DNA Isolation and Whole Genome Sequencing

Cultures of three *Spumella*-like species were established by a single cell isolation technique with glass pipetting from natural habitats: *Spumella* sp. Baeckdong012018B8 from freshwater, Jindo, Korea (34° 22’ 39” N, 126° 11’ 00” E); *Pedospumella* sp. Jangsampo120217C5 from the seashore, Taean, Korea (36° 25’ 24” N, 126° 21’ 33” E); and *Poteriospumella lacustris* Yongseonkyo072317C3 from freshwater, Gochang, Korea (35° 31’ 38” N, 126° 35’ 41” E). The three strains of *Spumella*-like flagellates are available from the Protist Culture Collection, Department of Biological Sciences, Chungnam National University, Korea. All cultures were grown in AF-6 medium (Andersen et al., 2005) with rice grains and distilled water for the freshwater strains or distilled seawater for the marine strain (*Pedospumella* sp. Jangsampo120217C5) and were maintained at 20°C under a 14:10 light/dark cycle with 30 µmol photons·m^−2^·s^−1^ from cool white fluorescent tubes.

All cultures were derived from single-cell isolates for unialgal cultivation. Total genomic DNA was extracted from exponentially growing cell cultures using the QIAGEN DNEasy Blood Mini Kit (QIAGEN, Valencia, CA, USA) following the manufacturer’s instructions. A paired-end library was prepared using the NexteraXT protocol (Illumina) according to the manufacturer’s protocol. Whole genome sequencing was performed using the Illumina MiSeq platform to generate paired-end 2 × 300 bp sequencing reads. More than 2 Gb of raw data were generated for each species.

### Genome Assembly and Annotation of Plastid Genomes

Sequence data were trimmed (i.e., base = 80 bp, error threshold = 0.05, n ambiguities = 2) using Trimmomatic 0.36 (Bolger et al., 2014) for *de novo* assembly with the default option (automatic bubble size, minimum contig length =1,000 bp). The trimmed reads were assembled using the SPAdes 3.7 assembler using *k-*mer size 127 (Bankevich et al., 2012) and then mapped using Geneious Pro 10.2.2 (Kearse et al., 2012) to assemble the contigs (similarity = 95%, length fraction = 75%), excluding contigs <1,000 bp. The assembled contigs were deemed to be of plastid genome origin as follows: (1) BLAST searches against the entire assembly using commonly known plastid genes as queries resulted in significant hits for these contigs (Jung et al., 2017) and (2) the predicted gene contents were similar to the previously published 160 kbp plastid genome of the chrysophytes *Ochromonas* sp. (KJ877675) and *Synura petersenii* (MH795128).

To aid in gene annotation, we created a database of protein-coding, rRNA, and tRNA genes using data from previously sequenced chrysophyte plastid genomes. Preliminary annotation of protein coding genes was performed using AGORA (Jung et al., 2018) and GeneMarkS (Besemer et al., 2001). The final annotation file was checked in Geneious Pro 10.2.2 (Kearse et al., 2012) using the ORF Finder (https://www.ncbi.nlm.nih.gov/orffinder/) with genetic code 11 (Bacterial, Archaeal, and Plant plastid code). The predicted open reading frames (ORFs) were checked manually, and the corresponding ORFs (and predicted functional domains) in the genome sequence were annotated.

The tRNA genes were identified using tRNAscan-SE version 1.21 (Lowe and Chan, 2016) with the default settings using the “Mito/Chloroplast” model. To help identify rRNA gene sequences, a set of known plastid rRNA sequences from the public database was used as a query sequence to search our new genome data using BLASTn. Physical maps were visualized with OrganellarGenomeDRAW 1.3.1 (Greiner et al., 2019). For structural and synteny comparisons, the genomes were aligned using GeneCo (Jung et al., 2019) with default settings.

### Phylogenomic and Phylogenetic Analyses

Phylogenomic analysis was carried out on amino acid sequence datasets created by combining 40 protein coding genes (8,267 amino acids) from 98 plastid genomes of stramenopiles. The plastid genome of *Hydrurus foetidus* was analyzed and annotated based on publicly available data on scaffold UYFQ01001012 (Bråte et al., 2019). The sequences of six haptophyte species were used as outgroup taxa for rooting purposes. For the phylogeny of the nuclear-encoded SSU rDNA gene (1,591 nucleotides) from 177 chrysophyte taxa and *Leukarachnion* sp., *Nannochloropsis limnetica*, and *Synchroma grande* were used as outgroup taxa. The dataset was aligned using MUSCLE 8.0 in the program MacGDE2.6 (Smith et al., 1994; Edgar, 2004).

Bayesian analyses were run using MrBayes 3.2.7 (Ronquist et al., 2012) with a random starting tree, two simultaneous runs (nruns = 2) and four Metropolis-coupled Markov chain Monte Carlo (MC3) algorithms for 2 x 10^7^ generations, with one tree retained every 1,000 generations. The burn-in point was identified graphically by tracking the likelihood values using TRACER v. 1.6 (http://tree.bio.ed.ac.uk/software/tracer/). ML phylogenetic analyses of individual protein alignments and concatenated alignments were conducted using IQ-TREE v.1.5.2 (Nguyen et al., 2015) with 1,000 bootstrap replicates. ML phylogenetic analysis of the nuclear-encoded SSU rDNA gene was performed using RAxML 8.1.20 (Stamatakis, 2014) with the general time reversible plus Gamma (GTR + GAMMA) model. We used 1,000 independent tree inferences using the -# option of the program to identify the best tree. The best evolutionary model for each tree was selected using the posterior mean site frequency (PMSF) model (the LG+F+G tree as the guide tree) incorporated in IQ-TREE. Trees were visualized using FigTree v.1.4.2 (http://tree.bio.ed.ac.uk/software/figtree/).

## Results and Discussion

### General Features of Non-Photosynthetic Chrysophyte Plastid Genomes

**Figure 1:**
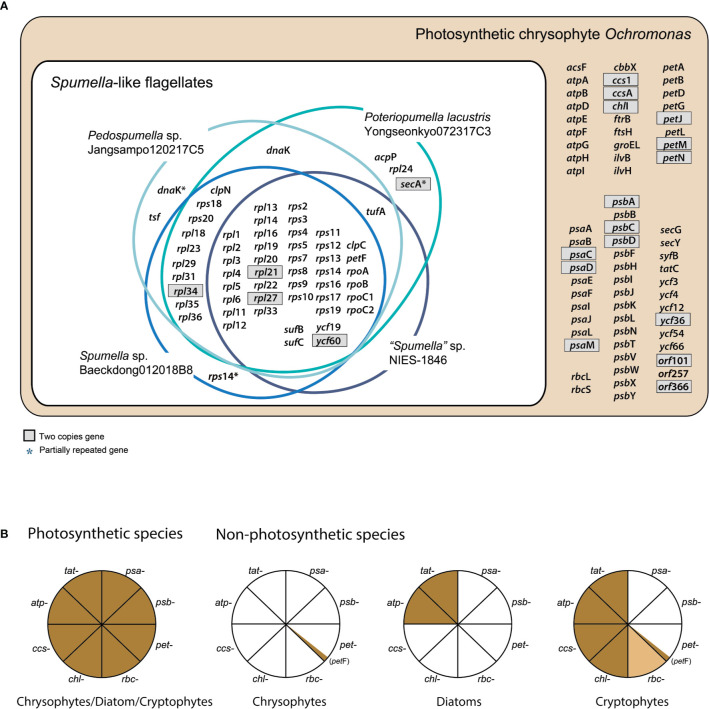
**(A)** Chrysophyte plastid genome content. Genes with two copies are shown in gray boxes. **(B)** Presence or absence of genes for photosystem I (PSI, *psa*-), photosystem II (PSII, *psb*-), the cytochrome *b6/f* complex (*pet*-), carbon fixation (*rbc*-), chlorophyll biosynthesis (*chl*-), cytochrome *c* biogenesis proteins (*ccs*-), the ATP synthase subunits (*atp*-), and the TAT system (*tat*-) are shown for three distantly related lineages, i.e., photosynthetic and non-photosynthetic chrysophytes, diatoms, and cryptophytes. Genes present/absent in plastid genomes are shown in brown or white, respectively. The *rbc*- gene present only in certain species is colored light brown. The data were derived from previously published studies (Donaher et al., 2009; Kamikawa et al., 2015; Kim et al., 2017; Dorrell et al., 2019; Kim et al., 2019; Tanifuji et al., 2020; this study).

Three new plastid genomes were sequenced from the non-photosynthetic chrysophte genera *Spumella, Pedospumella* and *Poteriospumella* (**Table 1**). The structures and coding capacities of these genomes were compared to those of the published genomes of the related photosynthetic chrysophyte *Ochromonas* sp. CCMP1393 and synuralean species (Ševčíková et al., 2015; Kim et al., 2019) and the non-photosynthetic *“Spumella”* sp. NIES-1846 (Dorrell et al., 2019). Most obvious is the fact that the plastid genomes of non-photosynthetic chrysophyte *Spumella*-like flagellates have lost all of the photosynthesis-related genes found in their photosynthetic relatives (**Figure 1**, see below). The plastid genome sizes of the four non-photosynthetic chrysophyte taxa range from ~53.2 kbp (*“Spumella”* sp. NIES-1846) to ~62.9 kbp (*Poteriospumella lacustris*), and the overall GC content ranged from 21.5% (*“Spumella”* sp. NIES-1846) to 38.9% (*Spumella* sp. Baeckdong012018B8) (**Table 1**). These taxa share a core set of 45 protein-coding genes, two rRNA operons, and 32–34 tRNAs. All of the plastid genomes of *Spumella*-like flagellates analyzed herein have typical IR regions, short single-copy (SSC) regions and long single-copy (LSC) regions. Putative lateral gene transfers from other organisms were not detected in any of the newly sequenced genomes.

**Table 1:** Characteristics of chrysophyte plastid genomes analyzed in this study.

General characteristics	*Pedospumella* sp. Jangsampo120217C5	*Poteriospumella lacustris* Yongseonkyo072317C3	*Spumella* sp. Baeckdong012018B8	*“Spumella”* sp. NIES-1846	*Ochromonas* sp. CCMP1393	*Synura petersenii* S114.C7, CZ	*Synura sphagnicola* CNUKRJ2	*Synura uvella* CNUKR	*Mallomonas splendens* CCMP1872	*Neotessella volvocina* CCMP1871
**Nutrition**	Heterotroph	Heterotroph	Heterotroph	Heterotroph	Mixotroph	Phototroph	Phototroph	Phototroph	Phototroph	Phototroph
**Type of plastid**	Leucoplast	Leucoplast	Leucoplast	Leucoplast	Plastid	Plastid	Plastid	Plastid	Plastid	Plastid
**Key characteristics for genus classification**	Small, colorless, naked single cell, independent clade in nuclear SSU rDNA phylogeny	Small, colorless, naked single cell, independent clade in nuclear SSU rDNA phylogeny	Small, colorless, naked single cell, independent clade in nuclear SSU rDNA phylogeny	Small, colorless, naked cell, independent clade in nuclear SSU rDNA phylogeny	naked single cell	colonized cells covered with silica scale on each cell	colonized cells covered with silica scale on each cell	colonized cells covered with silica scale on each cell	single cell covered with silica scale	colonized cells covered with silica scale on colony
**Size (bp)**	58,577	62,910	55,641	53,209	126,746	133,059	129,699	133,257	146,918	130,705
**Inverted repeat (IR)**	8,531	8,078	8,283	5,603	22,906	23,151	22,505	23,691	31,611	24,064
**Small single-copy region**	153	2,144	1,548	1,297	805	1,135	1,191	2,939	711	2,432
**Large single-copy region**	41,362	44,610	37,527	40,706	80,129	85,622	83,498	82,936	82,985	80,145
**G+C (%)**	35	32.2	38.9	21.5	30.9	37.89	38.76	38.19	42.39	37.54
**Total gene** **(include RNAs)**	97	99	97	82	183	182	181	189	187	186
**No. of protein-coding genes**	60	63	59	45	144	144	144	151	150	149
**tRNAs**	33	32	34	33	33	34	33	34	33	33
**rRNA operons**	2	2	2	2	2	2	2	2	2	2
**Introns**	–	–	–	–	–	*trn*L	*trn*L	*trn*L	*trn*L	*trn*P, *trn*S
**Unknown ORFs**	1	2	3	3	7	8	3	9	11	9
**Pseudogene**	–	–	–	–	–	–	–	*trn*R	*trn*R	*trn*E
**Partial copied gene**	*dna*K	*sec*A	*rps14*	*-*	*-*	–	*dna*K	–	*-*	*dna*K
**Specific encoded genes**	*pet*F	*pet*F	*pet*F	*pet*F	–	*dna*B/*cem*A	*dna*B/*cem*A	*dna*B/*cem*A	*dna*B/*cem*A	*cem*A
**Missing gene**	*psa-, psb-, pet-*, ***acp*P,** *acs*F*, cbb*X*, ccs*1*, ccs*A*, chl*I*, ftr*B*, ftr*H*, gro*EL*, ilv*B*, ilv*H*, rbc*L*, rbc*S, ***rpl*24**, ***sec*A** *, sec*G*, sec*Y*, syf*B*, tat*C,	*psa-, psb-, pet-, acs*F*, cbb*X*, ccs*1*, ccs*A*, chl*I*, gro*EL*, ftr*B*, ftr*H*, ilv*B*, ilv*H*, rbc*L*, rbc*S*, sec*G*, syf*B*, tat*C, ***tsf***,	*psa-, psb-, pet-, acs*F, ***acp*P,** *cbb*X*, ccs*1*, ccs*A*, chl*I, ***dna*K** *, ftr*B*, ftr*H*, gro*EL*, ilv*B*, ilv*H*, rbc*L*, rbc*S, ***rpl*24**, ***sec*A** *, sec*G*, sec*Y*, syf*B*, tat*C, ***tsf, tuf*A**	*psa-, psb-, pet-, acs*F, ***acp*P,** *cbb*X*, chl*I*, ccs*1*, ccs*A, ***clp*N**, ***dna*K** *, ftr*B*, ftr*H*, gro*EL*, ilv*B*, ilv*H*, rbc*L*, rbc*S, ***rpl*18**, ***rpl*23**, ***rpl*24**, ***rpl*29**, ***rpl*31**, ***rpl*34**, ***rpl*35**, ***rpl*36*, rps*18*, rps*20,** ***sec*A** *, sec*G*, sec*Y*, syf*B*, tat*C, ***tsf***	–	*syf*B	*syf*B	–	–	–
**GenBank accession**	**MN935477**	**MN935478**	**MN935479**	AP019363	KJ877675	MH795128	MH795129	NC_040134	NC_040135	MH795132

Bold type indicates gene loss in specific lineages among non-photosynthetic chrysophytes.

### Photosynthesis-Related Genes

**Figure 2:**
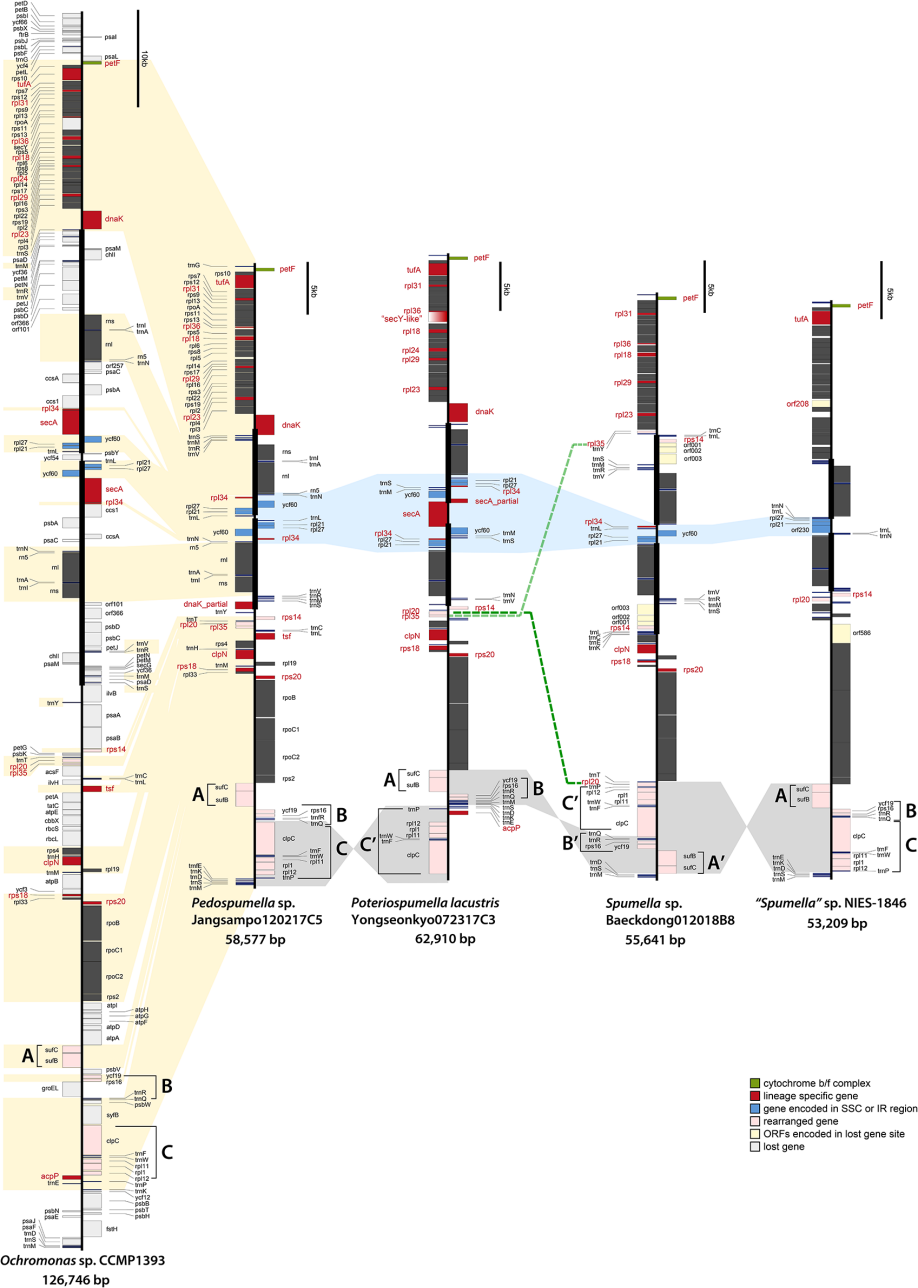
Linearized maps of the plastid genomes of chrysophytes. The structures and coding capacities of the non-photosynthetic chrysophyte plastid genomes examined herein are almost identical to those of the photosynthetic chrysophyte *Ochromonas* sp. CCMP1393. The protein-coding, rRNA and tRNA genes are labeled left or right of the line (genes on the left are transcribed bottom to top, those on the right top to bottom). Inverted repeats are highlighted with thick vertical lines (single-copy regions have thin lines). The gene clusters **(A–C)** are related to gene rearrangement.

Not surprisingly, the bulk of the gene loss in the plastid genomes of *Spumella*-like flagellates occurred in relation to photosynthesis (**Table 1**, **Figures 1** and **2**). This includes loss of genes for photosystem I, photosystem II, the cytochrome *b6/f* complex and ATP synthase subunits (**Figure 1**). Neither the genes for carbon fixation (RuBisCO) and chlorophyll biosynthesis (*chl*I) nor those encoding cytochrome *c* biogenesis proteins (*ccs*1 and *ccs*A) were found to be present in any of the non-photosynthetic chrysophyte plastid genomes.

The *psa* and *psb* gene families encode protein subunits of photosystem I and photosystem II, respectively. Although 10 *psa* genes and two photosystem I assembly protein genes (*ycf*3 and *ycf*4) occur in photosynthetic *Ochromonas* sp. CCMP1393 and synuralean species (Ševčíková et al., 2015; Kim et al., 2019), none are present in the plastid genomes of any *Spumella*-like flagellates. Similarly, the colorless cryptophyte plastid genome has lost all 11 *psa* genes found in the genomes of photosynthetic cryptophyte species (Donaher et al., 2009; Kim et al., 2017; Tanifuji et al., 2020), and the plastid genomes of non-photosynthetic diatoms (colorless *Nitzschia* spp.) have also lost all ten *psa* genes found in photosynthetic diatoms (Kamikawa et al., 2018). The photosynthetic chrysophyte plastid genomes possess a total of 16 *psb* genes, but all of these genes were absent in the four *Spumella*-like flagellates. The entire set of 18 *psb* genes has also been completely lost in non-photosynthetic cryptophytes and diatoms (**Figure 1B**).

The *pet* (photosynthetic electron transport) gene is almost completely absent from the plastid genomes of all non-photosynthetic *Spumella*-like flagellates, with the curious exception of *pet*F (**Figures 1A, B**). In photosynthetic organisms, *pet* proteins create a complex required for oxygenic photosynthesis, in particular the non-cyclic electron flow mediated by the cytochrome *b6f* complex at the thylakoid membrane. The *pet*F (ferredoxin) coding region of non-photosynthetic chrysophyte *Spumella*-like flagellates, as well as non-photosynthetic cryptophytes, appears to be intact and the predicted protein is surprisingly well conserved (**Figure 1B**). The gene may be involved in various redox reactions, given that transcriptome data from *Spumella*-like flagellates (Dorrell et al., 2019) show that genes for the proteins glutamine synthetase and glutamate synthase are expressed, although they do not contain obvious plastid-targeting sequences. In other secondarily non-photosynthetic organisms such as the colorless euglenoid *Euglena longa*, the *pet* genes are all missing (Gockel and Hachtel, 2000). In addition to *pet*F, the *Spumella*-like flagellates retain *suf*B/C (involved in Fe-S cluster biogenesis) in their plastid genomes.

Plastid genes involved in carbon fixation (RuBisCO subunit, *rbc*L and *rbc*S) and its regulation (*cbb*X), chlorophyll biosynthesis (*chl*I), and cytochrome *c* biogenesis proteins (*ccs*1 and *ccs*A) were absent in the *Spumella*-like flagellates but present in the plastids of photosynthetic chrysophytes. The cryptophytes represent an interesting point of comparison in that the *cbb*X, *rbc*L and *rbc*S genes are found in the genome of some colorless *Cryptomonas* species (e.g., *C.* *paramecium*) but not others (*Cryptomonas* spp. SAG9772f and CCAP1634B), while colorless diatoms appear to uniformly lack such genes (**Figure 1B**).

In contrast to photosynthetic chrysophytes, non-photosynthetic cryptophytes and diatoms, *Spumella*-like flagellates lack a full complement of plastid ATP synthase subunit genes, which are typically associated with the electron transport chain of photosynthesis (**Figure 1B**). The plastid genomes of non-photosynthetic cryptophytes contain eight ATP genes (*atp*A, B, D, E, F, G, H, and I), with the exception of an *atp*F pseudogene in *Cryptomonas paramecium* (Donaher et al., 2009; Tanifuji et al., 2020). Non-photosynthetic diatoms retain a near-complete set of ATP synthase genes in their plastid genomes, with *atp*E and *atp*F being present or absent in a species-specific fashion (Kamikawa et al., 2018). The loss of ATP synthase subunit genes in response to the loss of photosynthesis is a recurring theme in land plants and diatoms (Barrett et al., 2014; Kamikawa et al., 2018).

The ATP synthase complex is involved in generating proton gradients for thylakoid protein transporters, mediated by the twin arginine translocator (TAT) system, which depends on proton gradients to perform protein translocation across the thylakoid membrane. The *tat*C gene, the core protein of the TAT system, is absent from the genomes of the four *Spumella*-like flagellates compared here, whereas it is present in photosynthetic chrysophytes and non-photosynthetic cryptophytes and diatoms. With respect to plastid ultrastructure and ATP synthase complex genes, the *Spumella*-like flagellates we analyzed appear to have lost both thylakoid membranes and all of the ATP synthase complex genes that were present in their photosynthetic ancestors (**Figure 1B**). Interestingly, another *Spumella*-like flagellate, *Cornospumella* *fuschlensis* AR4D6, retains visible thylakoid structures (Grossmann et al., 2016). The colorless diatom *Nitzschia* sp. retains a few reduced thylakoid membranes in the plastid stroma and ATP synthase complex and TAT genes (Kamikawa et al., 2015), whereas the colorless cryptophyte *Cryptomonas paramecium* does not appear to possess any thylakoid membranes (Sepsenwol, 1973) but has retained many of its ATP synthase complex genes (Donaher et al., 2009; Tanifuji et al., 2020).

### Housekeeping Genes in Non-Photosynthetic Chrysophytes

The ribosomal protein operons in the four analyzed *Spumella*-like flagellate plastid genomes were found to be almost identical in terms of gene content and structure relative to each other and to those of the photosynthetic chrysophytes. 25 *rpl* genes for 50S ribosomal subunit proteins are found in photosynthetic chrysophytes, almost all of which are also present in the *Spumella*-like flagellate plastid genomes. The single exception is *rpl*24, which is absent in other *Spumella*-like flagellates but is present in *Poteriospumella* *lacustris* (**Figure 2**). The *rpl*24 gene has also been described as missing in the plastid genomes of Eustigmatophyceae, a stramenopile lineage that is closely related to chrysophytes based on plastid genome data (Ševčíková et al., 2019).

**Figure 3:**
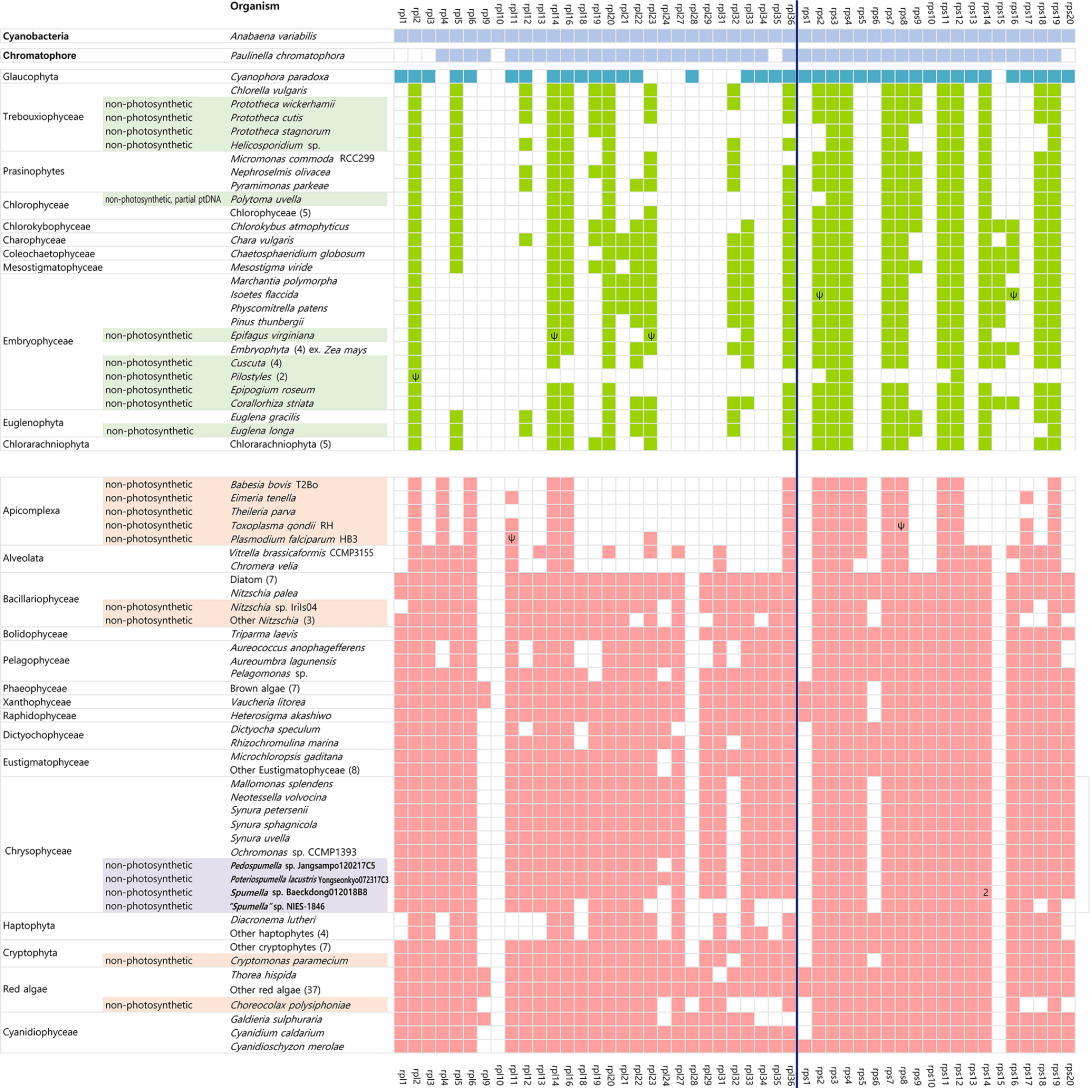
Presence/absence of plastid-encoded ribosomal protein genes in diverse algae and the cyanobacterium *Anabaena variabilis*. Taxa highlighted bold correspond to those specifically analyzed in this study. Filled boxes indicate the presence of ribosomal protein genes (green=green alga-derived secondary plastid lineage; red=red alga-derived plastid lineage). The missing ribosomal protein genes (i.e., *rpl*7, *rpl*8, *rpl*15, *rpl*17, *rpl*25, *rpl*26, *rpl*30, and *rps*21) were not detected in the plastid genomes of any of the lineages examined herein. Accession numbers and fully surveyed datasets are provided in **Supplementary Table S1**, online **Supplementary Material. 2**, multi-copy genes; Ψ, pseudogenes.

In terms of 30S ribosomal protein genes, seventeen are present in the plastid genomes of *Spumella*-like flagellates and photosynthetic chrysophyte species, with the exception of *rps*8 and *rps*20, which are missing in “*Spumella*” sp. NIES-1846. The *rps*14 gene was found to be duplicated and translocated in the genome of *Spumella* sp. Baeckdong012018B8 sequenced herein, whereas the previously published genome of “*Spumella*” sp. NIES-1846 is quite different from the three genomes we sequenced in terms of its *rpl* gene repertoire. No fewer than ten ribosomal protein genes were lost in the NIES-1846 strain: *rpl*18, *rpl*23, *rpl*24, *rpl*29, *rpl*31, *rpl*34, *rpl*35, *rpl*36, *rps*18, and *rps*20 (**Figures 2** and **3**). Six of these *rpl* gene losses (*rpl*18, *rpl*23, *rpl*24, *rpl*29, *rpl*31, and *rpl*36) map to the conserved ribosomal protein operon.

The set of ribosomal protein genes is generally conserved amongst the plastid genomes of diverse species, speaking to their importance in the assembly of functional organellar ribosomes. The smallest known number of plastid ribosomal protein genes occurs in apicomplexan apicoplasts: between 15 and 17 in total (Wilson and Williamson, 1997). In other non-photosynthetic organisms, the number of plastid-encoded ribosomal protein genes are only slightly reduced relative to phototrophs, such as in *Epifagus virginiana* (Wolfe et al., 1992) and the parasitic green alga species *Helicosporidium* sp. (de Koning and Keeling, 2004), and in non-photosynthetic diatoms, chrysophytes, cryptophytes, and parasitic red algae (**Figure 3** and **Supplementary Table S1**). For reasons that are unclear, the loss of ribosomal protein genes goes hand in hand with plastid genome reduction in non-photosynthetic species.

The *tuf*A gene, which encodes the plastid protein synthesis elongation factor Tu (EF-Tu), is present in almost all sequenced plastid genomes. In the green line, *tuf*A is present in the plastid genomes of most green algae, including the non-photosynthetic *Helicosporidium* and *Polytoma* species, but has been transferred to the nucleus in embryophytes and the charophycean *Zygnema circumcarinatum* (Martin et al., 1998; de Vries et al., 2013). In red alga-derived secondary plastids, *tuf*A is present in all sequenced genomes, even in apicomplexans and the non-photosynthetic diatom *Nitzschia* species, the only apparent exception being peridinin-pigmented dinoflagellates, which have extremely reduced plastid genomes (Howe et al., 2008). With the exception of *Spumella* sp. Baeckdong012018B8, all sequenced chrysophyte plastid genomes harbor the *tuf*A gene (**Figure 2**). While the *tuf*A gene was found to be missing in the plastid genome of *Spumella* sp. Baeckdong012018B8, gene transcripts for a putatively plastid-targeted EF-Tu protein were detected in transcriptome data from members of the core *Spumella* clade (Dorrell et al., 2019).

The molecular chaperone protein-coding genes *dna*K (a member of the hsp70 family) and *gro*EL (a chaperonin gene) are found in almost all red alga-derived plastids (i.e., those of cryptophytes, haptophytes, stramenopiles), as well as rhodophytes and glaucophytes (Green, 2011; Kim et al., 2017). In non-photosynthetic species, the colorless cryptophytes retain both *dna*K and *gro*EL, whereas colorless diatoms have only the *dna*K gene in their plastid genome (Dorrell et al., 2019; Tanifuji et al., 2020). The non-photosynthetic chrysophyte *Spumella*-like flagellate plastid genomes have uniformly lost the *gro*EL gene as well (**Table 1**), while the *dna*K gene shows a complex pattern of presence and absence in the four genomes we analyzed. The *dna*K gene is located in the IR region and has been partially copied in both IR regions in *Pedospumella* sp. Jangsampo120217C5. The partially copied *dna*K gene has also been detected in photosynthetic synuralean algae in a species-specific fashion (Kim et al., 2019). Here we found that the *dna*K gene is present in *Poteriospumella lacustris* Yongseonkyo072317C3 but is absent in *Spumella* sp. Baeckdong012018B8 and the “*Spumella”* sp. NIES-1846 (**Figure 2**).

The *sec*A gene encodes a protein translocase subunit involved in the hydrolysis of ATP to transfer proteins into the thylakoid lumen. The *sec*A gene is known to be absent in the glaucophytes, land plants and green algae, but is present in rhodophytes and the red alga-derived plastid genomes of cryptophytes, haptophytes, and stramenopiles. Colorless cryptophytes and diatoms (*Nitzschia* spp.) possess the gene (Dorrell et al., 2019; Tanifuji et al., 2020), but “*Spumella*” sp. NIES-1846 has lost it. In phototrophic chrysophytes, the *sec*A gene is present in the SSC region of the plastid genomes of *Ochromonas* sp. CCMP1393 and synuralean species (Kim et al., 2019). In the non-photosynthetic chrysophytes, we found the *sec*A gene to be present in *Poteriospumella* *lacustris* but absent in the plastid genomes of other *Spumella*-like flagellates; this gene absence correlates with extensive gene reduction in the SSC region (**Figure 2**). Interestingly, a *sec*Y-like gene was found between *rpl*36 and *rpl*18 in *Poteriospumella* *lacustris* (**Figure 2**, gradient red box). The Sec translocon subunits SecA and SecY, may function together for protein translocation across the thylakoid membranes. The existence of a divergent *sec*Y gene in *Poteriospumella lacustris* is consistent with the idea of ongoing plastid genome reduction in these organisms.

Previous studies have shown that the *tsf* gene, which encodes elongation factor Ts, is present in primary red algal plastids (Grzebyk et al., 2003). While photosynthetic chrysophytes also have a *tsf* gene in their plastid genomes, among the heterotrophic *Spumella*-like flagellates the gene was found to be present only in *Pedospumella* sp. Jangsampo120217C5; it does not reside in the plastid genomes of the other *Spumella*-like species examined in our study (**Figure 2**). The *tsf* gene was also not detected in transcriptome data from *Poteriospumella lacustris* (strains JBC07 and JBM10), *Pedospumella* *encystans* and *Pedospumella sinomuralis* (Dorrell et al., 2019).

Finally, the *acp*P gene, which encodes an acyl carrier protein, thus far appears to be present only in the plastid genome of *Poteriospumella* *lacustris* and photosynthetic chrysophytes (Ševčíková et al., 2015; Kim et al., 2019) (**Figure 2**). The acyl carrier protein is involved in the fatty acid biosynthesis pathway and is variably present and absent in the plastid genomes of other algae across the eukaryotic tree of life (Grzebyk et al., 2003).

### Plastid Genome Reduction and Rearrangement in Non-Photosynthetic Chrysophytes

A genome-wide comparison of synteny shows that plastid gene order in the *Spumella*-like flagellates is almost identical to that of photosynthetic chrysophytes (**Figure 2**), with the complete absence of the photosynthesis-related genes accounting for the bulk of the differences in genome size. We also carried out comparative genomic studies of plastid housekeeping genes in non-photosynthetic species among the red alga-derived plastids, including the “apicoplast” genome of the malaria parasite *Plasmodium falciparum* (Wilson et al., 1996; Waller and McFadden, 2005), colorless cryptophyte *Cryptomonas* species (Donaher et al., 2009; Tanifuji et al., 2020), and the colorless diatom *Nitzschia* sp. (Kamikawa et al., 2018). Overall, the presence of shared plastid genes in chrysophyte lineages reveals non-random retention of genes in the plastid genome despite the independent loss of photosynthesis in the four non-photosynthetic chrysophytes examined herein. Our data support the idea that independently evolved non-photosynthetic plastids show similar genome structure and gene loss patterns to those in the non-photosynthetic chrysophyte plastid genome.

The non-photosynthetic chrysophyte plastid genomes exhibit slightly different gene contents and structures in their inverted repeat (IR) regions (**Figure 2**). The IRs, which ranged in length from 8.08 kbp (*Poteriospumella lacustris*) to 8.53 kbp (*Pedospumella* sp. Jangsampo120217C5), contained three ribosomal protein genes (*rpl*21, *rpl*27, and *rpl*34), *ycf*60, three rRNAs and eight tRNAs. Twelve protein-coding genes in the repeat region were lost in the plastid genome of *Spumella*-like flagellates relative to the photosynthetic chrysophyte *Ochromonas* sp. CCMP1393 and synuralean plastid genomes (**Figure 2**).

The plastid genomes of the *Spumella*-like flagellates exhibit different gene orders and gene loss patterns among the three ribosomal proteins (*rpl*21, *rpl*27, and *rpl*34) and *ycf*60 in the IR/SSC junctions (**Figure 2**, marked in blue). The gene order in this region of the *Pedospumella* sp. Jangsampo120217C5 genome was found to be most similar to that of the photosynthetic chrysophyte *Ochromonas* sp. CCMP1393 and the synuralean species (i.e., loss of the *sec*A gene and the following gene order: *trn*N-*rpl*34-*ycf*60-*rpl*27-*rpl*21-*trn*L; **Figure 2**). The genes in this region of the *Poteriospumella* *lacustris* genome were inverted (*trn*L-*rpl*21-*rpl*27-*rpl*34-*trn*S-*trn*M-*ycf*60-partial *sec*A) and also contained a *sec*A gene in the SSC region. The *Spumella* sp. Baeckdong012018B8 genome was significantly reduced in this area, with the *rpl*34-*trn*L-*ycf*60-*rpl*27-*rpl*21 genes located in the SSC region. Finally, in the plastid genome of “*Spumella*” sp. NIES-1846, only the *trn*N-*trn*L genes were encoded in the IR region, with the *rpl*27-*rpl*21-*orf*230 genes being located in the SSC portion of the genome (and, as noted above, the *rpl*34 gene was lost).

Previous studies have shown that expansions and contractions are common in the IR boundaries of diatom and green algal genomes (Goulding et al., 1996; Wang et al., 2008; Jansen and Ruhlman, 2012; Sabir et al., 2014; Turmel et al., 2015). Here we have observed that instances of SSC/IR expansion and contraction have also occurred during the evolutionary history of non-photosynthetic *Spumella*-like flagellates, leading to changes in genome structure and coding capacity. Such events can alter gene order through inversion, transposition, gene loss, and/or contraction of the IR region. Such dynamics have also been observed in the LSC/IR junction of the plastid genomes of photosynthetic synuraleans, leading to gene rearrangements, expansion/contraction of the IR region, and gene loss events (Kim et al., 2019). The contraction of the IR region caused by gene loss is also likely one of the factors contributing to plastid genome reduction in non-photosynthetic chrysophytes.

### Gene Rearrangements

The *Spumella*-like flagellate plastid genomes are generally highly syntenic, with the exception of variations in the IR/SSC boundary regions and relative positions of 13 genes located in three clusters designated A, B and C (**Figure 2**). These clusters are as follows: (A) *suf*C-*suf*B, (B) *ycf*19-*rps*16-*trn*R-*trn*Q, and (C) *clp*C-*trn*F-*trn*W-*rpl*11-*rpl*1-*rpl*12-*trn*P. Three different cluster patterns were inferred. The first pattern, observed in *Pedospumella* sp. Jangsampo120217C5 and “*Spumella*” sp. NIES-1846, involves the clusters in the order A-B-C, and is shared with photosynthetic *Ochromonas* sp. CCMP1393 and synuralean species. The second pattern, A-B-C′, is observed in *Poteriospumella* *lacustris*, and the third pattern is seen in *Spumella* sp. Baeckdong012018B8; in this case the cluster order is C′-B′-A′ and is inverted relative to *Pedospumella* sp. Jangsampo120217C5 and “*Spumella*” sp. NIES-1846. The genes in these three clusters may be translocated and rearranged as a result of the loss of genes flanking the clusters in each species.

### Phylogenetic Relationships Among Chrysophytes

**Figure 4:**
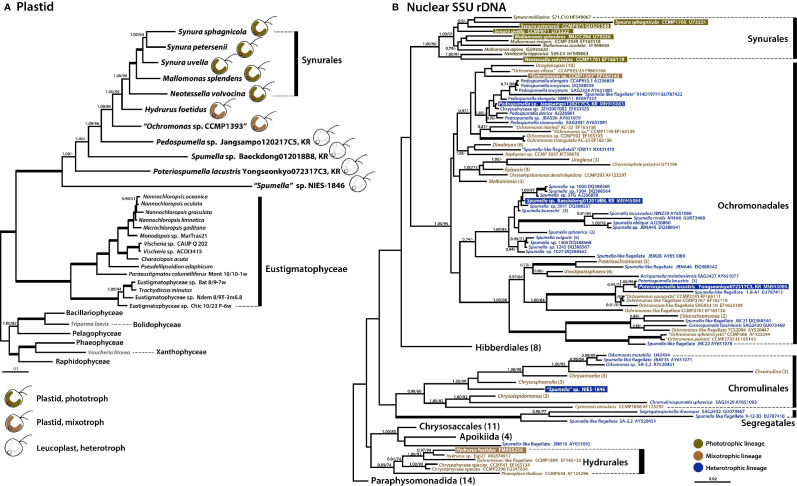
Phylogenies of phototrophic, mixotrophic, and heterotrophic chrysophytes. **(A)** Plastid genome-based Bayesian tree of chrysophytes and other stramenopile taxa. The topology of this tree (inferred from an alignment of 40 proteins and 8,267 amino acids) is consistent with a single acquisition of photosynthetic ability from a red alga-derived secondary plastid in a chrysophyte ancestor. The numbers on each node represent posterior probabilities (left) and ultrafast bootstrap approximation (UFBoot) values calculated using IQ-Tree (right). Thick branches indicate fully supported nodes (PP = 1.00/ML = 100). This tree shows the phylogenetic relationships of chrysophytes and other stramenopiles based on a subset of taxa; for phylogenies inferred using a fully expanded dataset, refer to **Supplementary Figures S1** and **S3**. **(B)** Nuclear SSU rDNA tree of chrysophytes showing the putative relationships of the phototrophic chrysophyte lineage in the context of non-photosynthetic lineages. Sequences from the strains whose plastid genomes were sequenced in this study are highlighted. Together, the plastid and nuclear gene tree topologies suggest parallel evolution of chrysophyte plastid genomes in response to shifts to heterotrophy. The numbers on each node represent posterior probabilities (left) and maximum-likelihood (ML) bootstrap support values calculated using RAxML (right). Support values (PP < 0.70/ML<70) are shown on each node. Bold branches indicates fully supported values (PP = 1.00/ML = 100). The numbers in () are indicate the number of taxa in the species, genus, or order. The species name with “ “ indicates uncertain taxonomic status. An expanded phylogeny based on a much larger dataset is provided in **Supplementary Figure S2**. The scale bars indicate the number of substitutions/site.

Phylogenomic analysis using 40 plastid-encoded proteins showed a monophyletic assemblage of chrysophytes within stramenopiles with maximal support (posterior probability (PP)=1.00, maximum likelihood (ML)=100%, **Supplementary Table S2**). The chrysophyte assemblage formed a sister relationship with Eustigmatophyceae (**Figure 4A** and **Supplementary Figure S1**), consistent with recently published studies suggesting that eustigmatophyte plastids are closely related to those of chrysophytes (Ševčíková et al., 2015; Han et al., 2019; Kim et al., 2019; Ševčíková et al., 2019). Our plastid phylogenomic investigations showed that the mixotrophic species *Ochromonas* sp. CCMP1393 and the putatively facultative photoautotrophic species *Hydrurus foetidus* formed a strongly supported sister relationship with the exclusively photosynthetic synuralean assemblage (**Figure 4A**), which is consistent with previous multigene phylogenetic studies based on plastid-encoded proteins (Kim et al., 2019).

We also carried out a phylogenetic analysis of nuclear SSU rDNA sequences from a wide range of chrysophyte species (**Figure 4B**) to serve as a reference point for interpreting the plastid genome tree and, more generally, to better understand changes in nutritional modes during the evolution of the group. The photosynthetic synuralean lineage was found to form a monophyletic assemblage. We also found that sequences from non-photosynthetic chrysophytes were intermixed with those of mixotrophic lineages (Grossmann et al., 2016; Kristiansen and Škaloud, 2017; Dorrell et al., 2019; this study). Collectively, the combined results of plastid and nuclear gene phylogenies demonstrate that photosynthesis has been lost multiple times during the evolutionary history of chrysophytes.

### The Biology and Evolution of Chrysophytes and Their Plastid Genomes

Photosynthetic chrysophytes are generally mixotrophs that rely on the ingestion of organic nutrients, with the exception of the phototrophic synuralean lineage (Graupner et al., 2018; Lie et al., 2018). The chrysophyte *Hydrurus foetidus* forms filamentous branches within a soft polysaccharide coat and is a putatively facultative phototrophic species (Klaveness and Lindstrøm, 2011; Lavoie et al., 2018). Genome size and cell size differ significantly among species exhibiting different nutritional modes (Olefeld et al., 2018). Where known, heterotrophic chrysophytes have the smallest genomes and cell volumes, phototrophs have the largest, and mixotrophs are generally in between the two. The extreme reduction in the plastid genome of the non-photosynthetic *Spumella*-like flagellates examined herein surely results from the evolution of a heterotrophic lifestyle concomitant with the loss of genes for photosynthesis that are no longer needed. According to Olefeld et al., 2018, the loss of photosynthesis in chrysophytes may be due to energetic factors. Chrysophytes generally do not have a carbon-concentrating mechanism (CCM), indicating that they do not use bicarbonate (HCO_3_^-^) as a carbon source in diverse water environments (Maberly et al., 2009) and that they are subject to carbon limitation as a selection pressure. Therefore, many chrysophyte lineages may have independently changed their nutritional mode to that of obligate heterotrophs.

**Figure 5:**
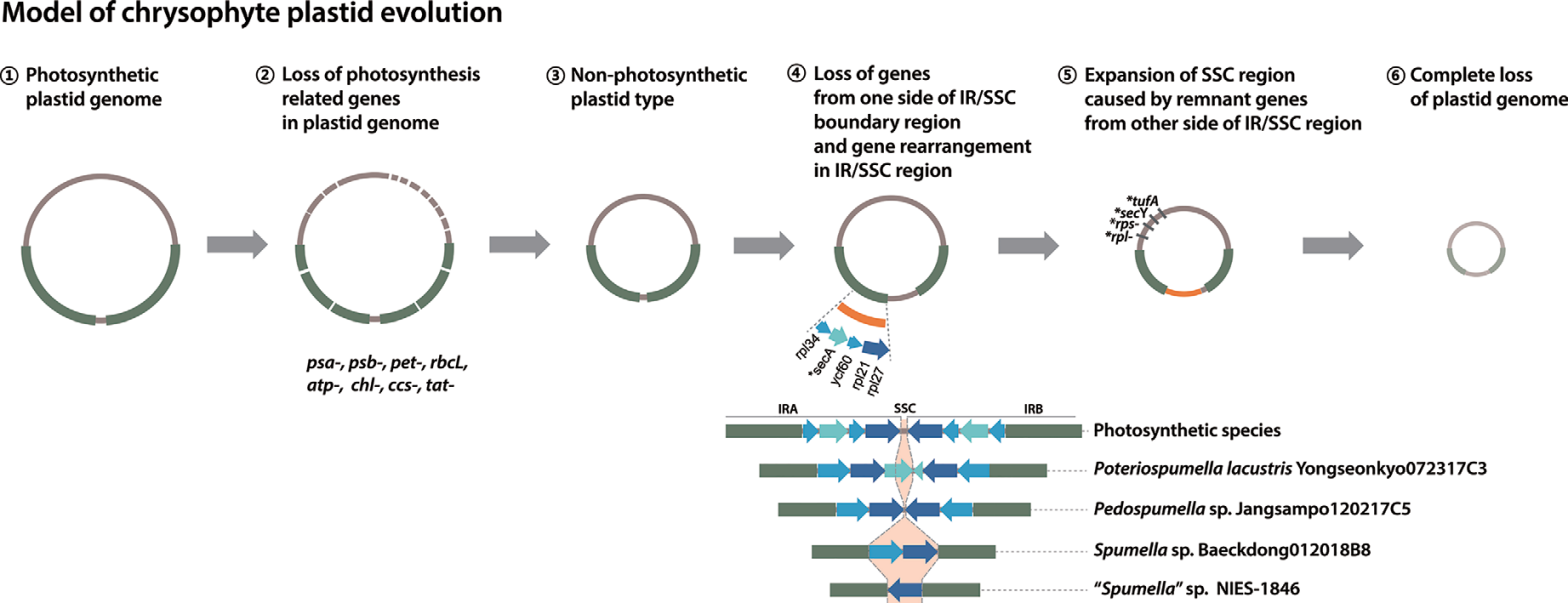
Model of chrysophyte plastid evolution. Lineage-specific gene losses in step ⑤ are indicated by a star (*).

The phylogenetic analyses carried out in our study and elsewhere (e.g., Dorrell et al., 2019) provide clear evidence for parallel evolution of plastid genomes in response to a shift to heterotrophy. How many times this occurred is still unclear. Nevertheless, from the data in hand we propose a modified model of chrysophyte plastid evolution, as follows (**Figure 5**). The red alga-type plastid of photosynthetic chrysophytes stems from a secondary (or possibly) tertiary endosymbiotic event in an ancestor shared with other plastid-bearing stramenopiles (see Sibbald and Archibald, 2020 and references therein; **Figure 5**, ①). Much later, after the diversification of chrysophytes, *Spumella*-like flagellates lost their photosynthesis-related genes on multiple occasions (**Figure 5**, ②, ③). Plastid genome reduction occurred as a result of gene loss from one side of the IR/SSC boundary region (**Figure 5**, ④), and the expansion of the SSC region led to the presence of remnant genes on the other side of the IR/SSC region (**Figure 5**, ⑤). The loss of much larger numbers of genes brought about complete loss of plastid genomes (**Figure 5**, ⑥), as observed in the paraphysomonad clade (Dorrell et al., 2019). The loss of the *sec*A, *sec*Y, *tuf*A, *rpl*, and *rps* genes in a species-specific fashion and contraction of the IR/SSC boundary region observed in our study provide examples of genome reduction “in action,” providing evidence consistent with this model.

## Conclusions

Analysis of three newly sequenced plastid genomes from *Spumella*-like flagellates has provided insight into the fine-scale dynamics of genome reduction in chrysophytes. Heterotrophic lineages appear to have evolved from photosynthetic and mixotrophic ones multiple times independently during chrysophyte evolution. Our results are consistent with previous suggestions that heterotrophic chrysophytes are polyphyletic and their plastid genomes have undergone extreme reduction due to the loss of photosynthesis-related genes. The almost identical gene content and structure of the genomes of *Spumella*-like flagellates analyzed herein suggests that non-photosynthetic chrysophytes have experienced parallel gene losses as they independently transitioned from phototrophy to heterotrophy.

## Data Availability Statement

The plastid genome sequences were deposited in the NCBI GenBank database under the following accession numbers: MN935477 (*Pedospumella* sp. Jangsampo120217C5), MN935478 (*Poteriospumella lacustris*) and MN935479 (*Spumella* sp. Baeckdong012018B8). The nuclear SSU rDNA sequences of three Spumella-like flagellates were deposited in the NCBI GenBank database under the following accession numbers: MN945085 (*Pedospumella* sp. Jangsampo120217C5), MN945083 (*Poteriospumella lacustris* Yongseonkyo072317C3), and MN945084 (*Spumella* sp. Baeckdong012018B8).

## Author Contributions

JIK and WS conceived and designed the experiments. JIK and MJ performed the experiments and analyzed the data. JIK, JMA, and WS interpreted the data and wrote the manuscript. All authors contributed to the article and approved the submitted version.

## Funding

This study was supported by funds from the National Research Foundation (NRF) of Korea (NRF-2015R1D1A1A01057899 and 2018R1D1A1B07050727) provided to JIK, a Natural Sciences and Engineering Research Council of Canada grant (RGPIN-2014) awarded to JMA, and the Collaborative Genome Program funded by the Ministry of Oceans and Fisheries (M01201820180430) and the NRF (2019R1I1A2A01063159 and 2015M1A5A1041808) to WS. These funding organizations were not involved in the design of the study, the collection, analysis or interpretation of the data, or the writing of the manuscript.

## Conflict of Interest

The authors declare that the research was conducted in the absence of any commercial or financial relationships that could be construed as a potential conflict of interest.

## Supplementary Material

Additional file 1: Supplementary Figure S1 Phylogenetic tree of chrysophyte plastid-encoded proteins and those of other photosynthetic stramenopiles. This tree was constructed using a dataset of 40 concatenated protein-coding genes (8,297 amino acids) selected with a main focus on the leucoplasts of non-photosynthetic chrysophyte *Spumella*-like flagellates. The numbers on each node represent posterior probabilities (left) and ultrafast bootstrap approximation (UFBoot) values calculated using IQ-Tree (right). The bold branch indicates strongly supported values (PP = 1.00/ML = 100). The scale bar indicates the number of substitutions/site.

Additional file 2: Supplementary Figure S2 The nuclear encoded SSU rDNA tree of 177 chrysophytes and 3 outgroup taxa showing the putative relationships of the non-photosynthetic chrysophyte lineages relative to the photosynthetic ones. The NCBI accession numbers are provided with taxon names. The numbers on each node represent posterior probabilities using Bayesian analysis. The bold branch indicates strongly supported values (PP = 1.00). The scale bar indicates the number of substitutions/site.

Additional file 3: Supplementary Figure S3 Phylogenetic tree of chrysophyte plastids and those of other photosynthetic stramenopiles. This tree was constructed using a dataset of 40 concatenated protein-coding genes selected with a main focus on the leucoplasts of non-photosynthetic chrysophyte *Spumella*-like flagellates (8,297 amino acids). The tree was generated using the PMSF model (the LG+F+G tree as guide tree) and ultrafast bootstrap approximation (UFBoot) values calculated using IQ-Tree.
