# Supplementary figures and images for: Comparative Plastid Genomics of Non-Photosynthetic Chrysophytes: Genome Reduction and Compaction
Source: Front Plant Sci. 2020 Sep 10;11:572703. doi: 10.3389/fpls.2020.572703 (PMC7511666; doi:10.3389/fpls.2020.572703)

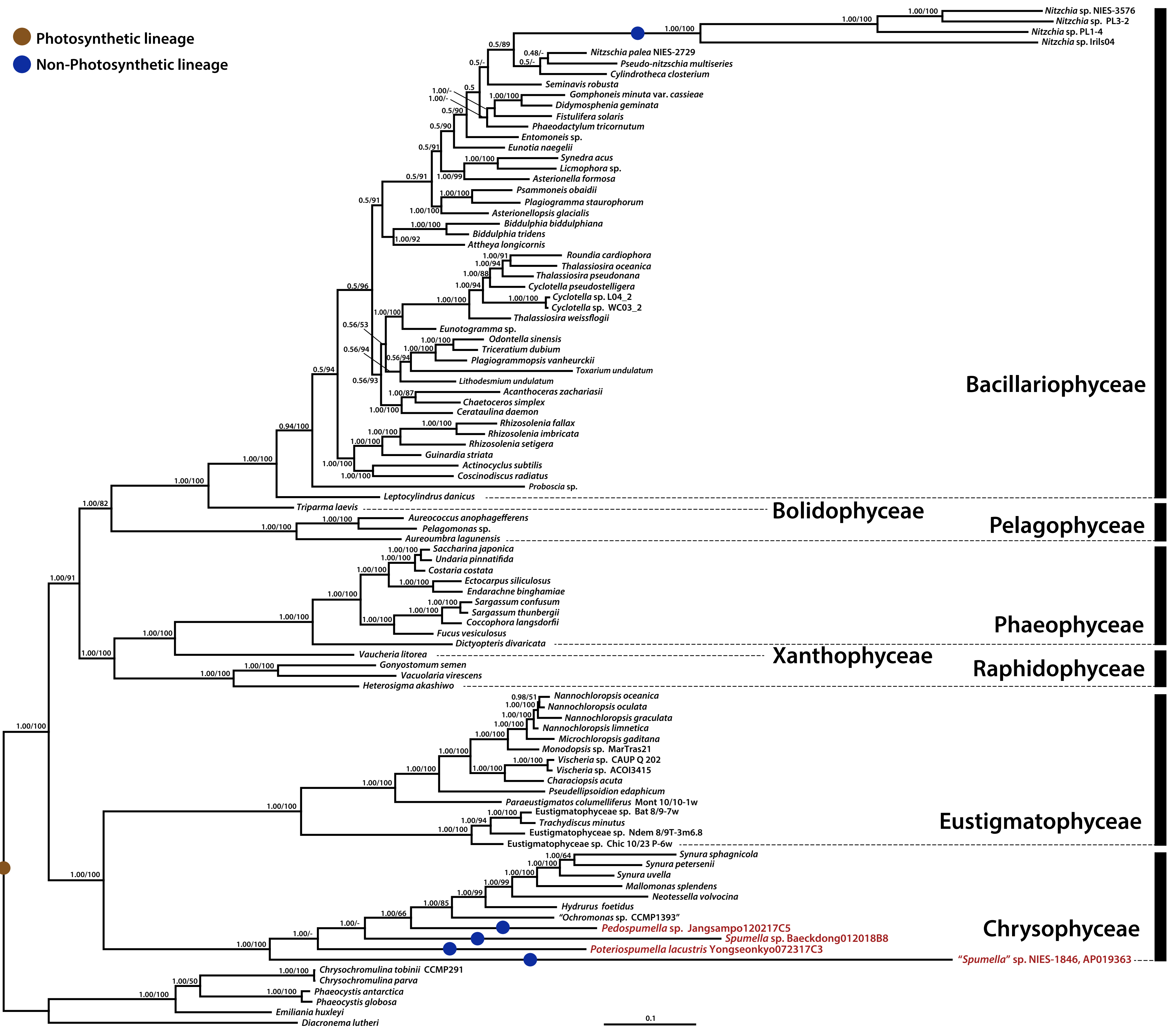

Supplement: Additional file 1: Supplementary Figure S1 — Phylogenetic tree of chrysophyte plastid-encoded proteins and those of other photosynthetic stramenopiles. This tree was constructed using a dataset of 40 concatenated protein-coding genes (8,297 amino acids) selected with a main focus on the leucoplasts of non-photosynthetic chrysophyte Spumella-like flagellates. The numbers on each node represent posterior probabilities (left) and ultrafast bootstrap approximation (UFBoot) values calculated using IQ-Tree (right). The bold branch indicates strongly supported values (PP = 1.00/ML = 100). The scale bar indicates the number of substitutions/site. [file DataSheet_1.pdf]
