# Supplementary material for: Comparative Plastid Genomics of Non-Photosynthetic Chrysophytes: Genome Reduction and Compaction
Source: Front Plant Sci. 2020 Sep 10;11:572703. doi: 10.3389/fpls.2020.572703 (PMC7511666; doi:10.3389/fpls.2020.572703)
Supplement: Additional file 2: Supplementary Figure S2 — The nuclear encoded SSU rDNA tree of 177 chrysophytes and 3 outgroup taxa showing the putative relationships of the non-photosynthetic chrysophyte lineages relative to the photosynthetic ones. The NCBI accession numbers are provided with taxon names. The numbers on each node represent posterior probabilities using Bayesian analysis. The bold branch indicates strongly supported values (PP = 1.00). The scale bar indicates the number of substitutions/site. [file DataSheet_2.pdf]

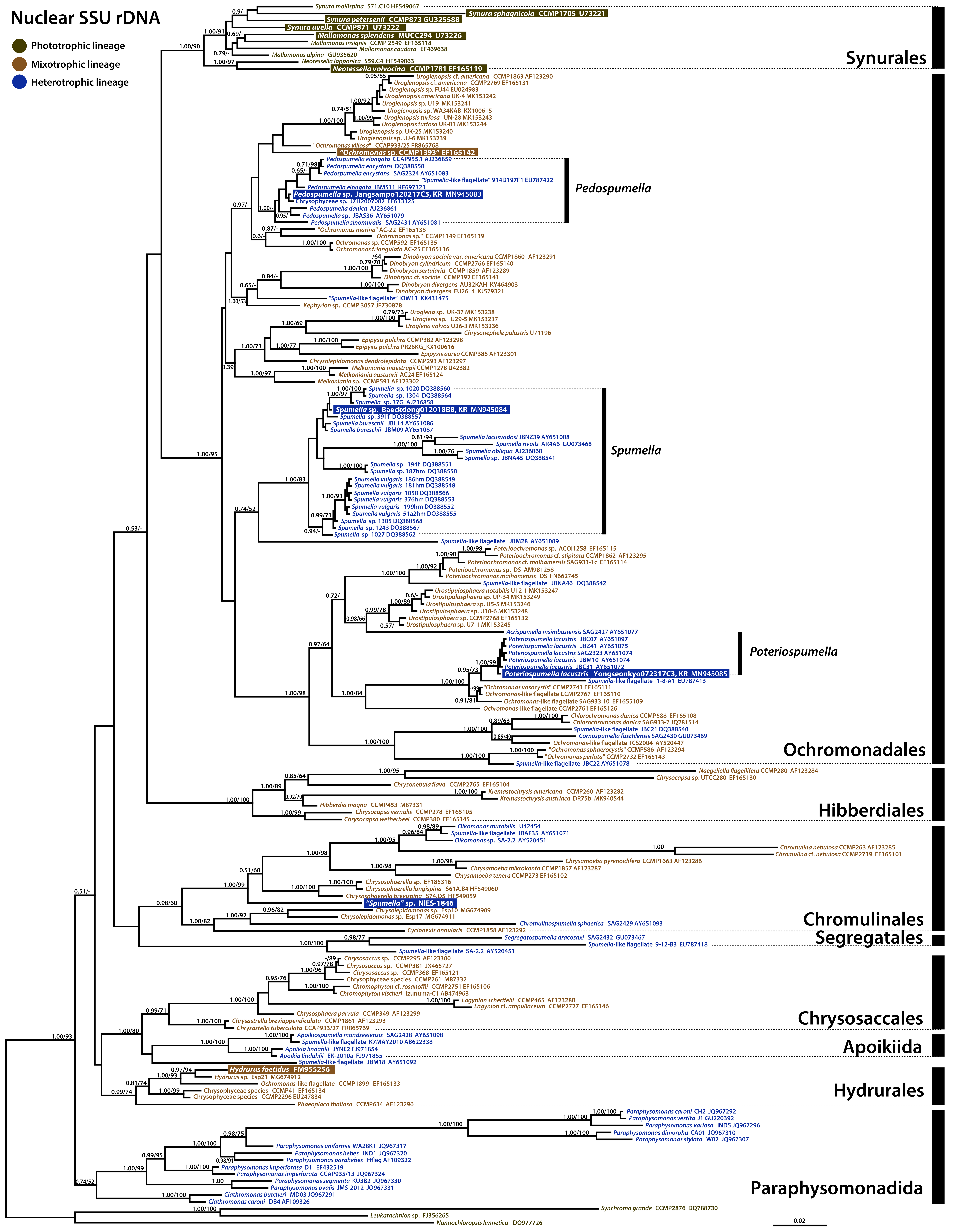

Nuclear SSU rDNA
Phototrophic lineage
Mixotrophic lineage
Heterotrophic lineage
Synura mollispina S71.C10 HF549067
Synura sphagnicola CCMP1705 U73221
Synura petersenii CCMP873 GU325588
Synura uvella CCMP871 U73222
Mallomonas splendens MUCC294 U73226
Mallomonas insignis CCMP 2549 EF165118
Mallomonas caudata EF469638
Mallomonas alpina GU935620
Neotessella lapponica S59.C4 HF549063
Neotessella volvocina CCMP1781 EF165119
Synurales
Uroglenopsis cf. americana CCMP1863 AF123290
Uroglenopsis cf. americana CCMP2769 EF165131
Uroglenopsis sp. FU44 EU024983
Uroglenopsis americana UK-4 MK153242
Uroglenopsis sp. U19 MK153241
Uroglenopsis sp. WA34KAB KX100615
Uroglenopsis turfosa UN-28 MK153243
Uroglenopsis turfosa UK-81 MK153244
Uroglenopsis sp. UK-25 MK153240
Uroglenopsis sp. UJ-6 MK153239
"Ochromonas villosa" CCAP933/25 FR865768
"Ochromonas sp. CCMP1393" EF165142
Pedospumella elongata CCAP955.1 AJ236859
Pedospumella encystans DQ388558
Pedospumella encystans SAG2324 AY651083
"Spumella-like flagellate" 914D197F1 EU787422
Pedospumella elongata JBMS11 KF697323
Pedospumella sp. Jangsampo120217C5, KR MN945083
Chrysophyceae sp. JZH2007002 EF633325
Pedospumella danica AJ236861
Pedospumella sp. JBAS36 AY651079
Pedospumella sinomuralis SAG2431 AY651081
Pedospumella
"Ochromonas marina" AC-22 EF165138
"Ochromonas sp." CCMP1149 EF165139
Ochromonas sp. CCMP592 EF165135
Ochromonas triangulata AC-25 EF165136
Dinobryon sociale var. americana CCMP1860 AF123291
Dinobryon cylindricum CCMP2766 EF165140
Dinobryon sertularia CCMP1859 AF123289
Dinobryon cf. sociale CCMP392 EF165141
Dinobryon divergens AU32KAH KY464903
Dinobryon divergens FU26_4 KJ579321
"Spumella-like flagellate" IOW11 KX431475
Kephyrion sp. CCMP 3057 JF730878
Uroglena sp. UK-37 MK153238
Uroglena sp. U29-5 MK153237
Uroglena volvox U26-3 MK153236
Chrysonephele palustris U71196
Epipyxis pulchra CCMP382 AF123298
Epipyxis pulchra PR26KG_KX100616
Epipyxis aurea CCMP385 AF123301
Chrysolepidomonas dendrolepidota CCMP293 AF123297
Melkoniania moestrupii CCMP1278 U42382
Melkoniania austuarii AC24 EF165124
Melkoniania sp. CCMP591 AF123302
Spumella sp. 1020 DQ388560
Spumella sp. 1304 DQ388564
Spumella sp. 37G AJ236858
Spumella sp. Baeckdong012018B8, KR MN945084
Spumella sp. 391f DQ388557
Spumella bureschii JBL14 AY651086
Spumella bureschii JBM09 AY651087
Spumella lacusvadosi JBNZ39 AY651088
Spumella rivalis AR4A6 GU073468
Spumella obliqua AJ236860
Spumella sp. JBNA45 DQ388541
Spumella sp. 194f DQ388551
Spumella sp. 187hm DQ388550
Spumella vulgaris 186hm DQ388549
Spumella vulgaris 181hm DQ388548
Spumella vulgaris 1058 DQ388566
Spumella vulgaris 376hm DQ388553
Spumella vulgaris 199hm DQ388552
Spumella vulgaris 51a2hm DQ388555
Spumella sp. 1305 DQ388568
Spumella sp. 1243 DQ388567
Spumella sp. 1027 DQ388562
Spumella
Spumella-like flagellate JBM28 AY651089
Poterioochromonas sp. ACOI1258 EF165115
Poterioochromonas cf. stipitata CCMP1862 AF123295
Poterioochromonas cf. malhamensis SAG933-1c EF165114
Poterioochromonas sp. DS AM981258
Poterioochromonas malhamensis DS FN662745
Spumella-like flagellate JBNA46 DQ388542
Urostipulosphaera notabilis U12-1 MK153247
Urostipulosphaera sp. UP-34 MK153249
Urostipulosphaera sp. U5-5 MK153246
Urostipulosphaera sp. U10-6 MK153248
Urostipulosphaera sp. CCMP2768 EF165132
Urostipulosphaera sp. U7-1 MK153245
Acrispumella msimbasiensis SAG2427 AY651077
Poteriospumella lacustris JBC07 AY651097
Poteriospumella lacustris JBZ41 AY651075
Poteriospumella lacustris SAG2323 AY651074
Poteriospumella lacustris JBM10 AY651074
Poteriospumella lacustris JBC31 AY651072
Poteriospumella lacustris Yongseonkyo072317C3, KR MN945085
Poteriospumella
Spumella-like flagellate 1-8-A1 EU787413
"Ochromonas vasocystis" CCMP2741 EF165111
Ochromonas-like flagellate CCMP2767 EF165110
Ochromonas-like flagellate SAG933.10 EF1655109
Ochromonas-like flagellate CCMP2761 EF165126
Chlorochromonas danica CCMP588 EF165108
Chlorochromonas danica SAG933-7 JQ281514
Spumella-like flagellate JBC21 DQ388540
Cornospumella fuschlensis SAG2430 GU073469
Ochromonas-like flagellate TCS2004 AY520447
"Ochromonas sphaerocystis" CCMP586 AF123294
"Ochromonas perlata" CCMP2732 EF165143
Spumella-like flagellate JBC22 AY651078
Ochromonadales
Naegeliella flagellifera CCMP280 AF123284
Chrysocapsa sp. UTCC280 EF165130
Chrysonebula flava CCMP2765 EF165104
Kremastochrysis americana CCMP260 AF123282
Kremastochrysis austriaca DR75b MK940544
Hibberdia magna CCMP453 M87331
Chrysocapsa vernalis CCMP278 EF165105
Chrysocapsa wetherbeei CCMP380 EF165145
Hibberdiales
Oikomonas mutabilis U42454
Spumella-like flagellate JBAF35 AY651071
Oikomonas sp. SA-2.2 AY520451
Chromulina nebulosa CCMP263 AF123285
Chromulina cf. nebulosa CCMP2719 EF165101
Chrysamoeba pyrenoidifera CCMP1663 AF123286
Chrysamoeba mikrokonta CCMP1857 AF123287
Chrysamoeba tenera CCMP273 EF165102
Chrysosphaerella sp. EF185316
Chrysosphaerella longispina S61A.B4 HF549060
Chrysosphaerella brevispina S74.D5 HF549059
"Spumella" sp. NIES-1846
Chrysolepidomonas sp. Esp10 MG674909
Chrysolepidomonas sp. Esp17 MG674911
Chromulinospumella sphaerica SAG2429 AY651093
Cyclonexis annularis CCMP1858 AF123292
Chromulinales
Segregatospumella dracosaxi SAG2432 GU073467
Spumella-like flagellate 9-12-B3 EU787418
Spumella-like flagellate SA-2.2 AY520451
Segregatales
Chrysosaccus sp. CCMP295 AF123300
Chrysosaccus sp. CCMP381 JX465727
Chrysosaccus sp. CCMP368 EF165121
Chrysophyceae species CCMP261 M87332
Chromophyton cf. rosanoffii CCMP2751 EF165106
Chromophyton vischeri Izunuma-C1 AB474963
Lagynion scherffelii CCMP465 AF123288
Lagynion cf. ampullaceum CCMP2727 EF165146
Chrysosphaera parvula CCMP349 AF123299
Chrysastrella breviappendiculata CCMP1861 AF123293
Chrysastella tuberculata CCAP933/27 FR865769
Chrysosaccales
Apoikiospumella mondseeiensis SAG2428 AY651098
Spumella-like flagellate K7MAY2010 AB622338
Apoikia lindahlii JYNE2 FJ971854
Apoikia lindahlii EK-2010a FJ971855
Spumella-like flagellate JBM18 AY651092
Apoikiida
Hydrurus foetidus FM955256
Hydrurus sp. Esp21 MG674912
Ochromonas-like flagellate CCMP1899 EF165133
Chrysophyceae species CCMP41 EF165134
Chrysophyceae species CCMP2296 EU247834
Phaeoplaca thallosa CCMP634 AF123296
Hydrurales
Paraphysomonas caroni CH2 JQ967292
Paraphysomonas vestita J1 GU220392
Paraphysomonas variosa IND5 JQ967296
Paraphysomonas dimorpha CA01 JQ967310
Paraphysomonas stylata W02 JQ967307
Paraphysomonas uniformis WA28KT JQ967317
Paraphysomonas hebes IND1 JQ967320
Paraphysomonas parahebes Hflag AF109322
Paraphysomonas imperforata D1 EF432519
Paraphysomonas imperforata CCAP935/13 JQ967324
Paraphysomonas segmenta KU3B2 JQ967330
Paraphysomonas ovalis JMS-2012 JQ967331
Clathromonas butcheri MD03 JQ967291
Clathromonas caroni DB4 AF109326
Paraphysomonadida
Synchroma grande CCMP2876 DQ788730
Leukarachnion sp. FJ356265
Nannochloropsis limnetica DQ977726
0.02
