# Supplementary material for: Comparative Plastid Genomics of Non-Photosynthetic Chrysophytes: Genome Reduction and Compaction
Source: Front Plant Sci. 2020 Sep 10;11:572703. doi: 10.3389/fpls.2020.572703 (PMC7511666; doi:10.3389/fpls.2020.572703)
Supplement: Additional file 3: Supplementary Figure S3 — Phylogenetic tree of chrysophyte plastids and those of other photosynthetic stramenopiles. This tree was constructed using a dataset of 40 concatenated protein-coding genes selected with a main focus on the leucoplasts of non-photosynthetic chrysophyte Spumella-like flagellates (8,297 amino acids). The tree was generated using the PMSF model (the LG+F+G tree as guide tree) and ultrafast bootstrap approximation (UFBoot) values calculated using IQ-Tree. [file DataSheet_3.pdf]

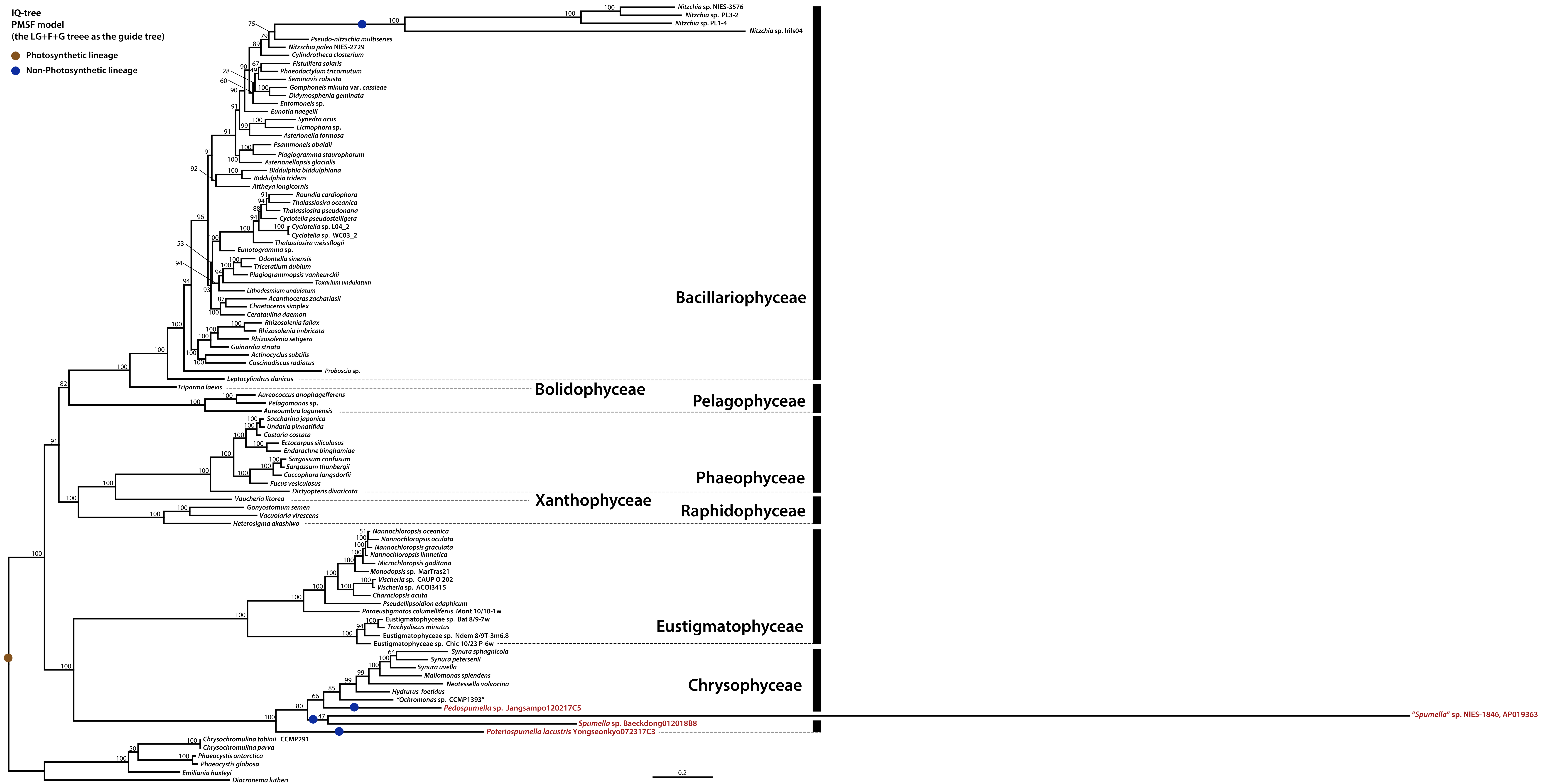

IQ-tree
PMSF model
(the LG+F+G treee as the guide tree)
Photosynthetic lineage
Non-Photosynthetic lineage
Nitzchia sp. NIES-3576
Nitzchia sp. PL3-2
Nitzchia sp. PL1-4
Nitzchia sp. Irils04
Pseudo-nitzschia multiseries
Nitzschia palea NIES-2729
Cylindrotheca closterium
Fistulifera solaris
Phaeodactylum tricornutum
Seminavis robusta
Gomphoneis minuta var. cassieae
Didymosphenia geminata
Entomoneis sp.
Eunotia naegelii
Synedra acus
Licmophora sp.
Asterionella formosa
Psammoneis obaidii
Plagiogramma staurophorum
Asterionellopsis glacialis
Biddulphia biddulphiana
Biddulphia tridens
Attheya longicornis
Roundia cardiophora
Thalassiosira oceanica
Thalassiosira pseudonana
Cyclotella pseudostelligera
Cyclotella sp. L04_2
Cyclotella sp. WC03_2
Thalassiosira weissflogii
Eunotogramma sp.
Odontella sinensis
Triceratium dubium
Plagiogrammopsis vanheurckii
Toxarium undulatum
Lithodesmium undulatum
Acanthoceras zachariasii
Chaetoceros simplex
Cerataulina daemon
Rhizosolenia fallax
Rhizosolenia imbricata
Rhizosolenia setigera
Guinardia striata
Actinocyclus subtilis
Coscinodiscus radiatus
Proboscia sp.
Leptocylindrus danicus
Triparma laevis
Aureococcus anophagefferens
Pelagomonas sp.
Aureoumbra lagunensis
Saccharina japonica
Undaria pinnatifida
Costaria costata
Ectocarpus siliculosus
Endarachne binghamiae
Sargassum confusum
Sargassum thunbergii
Coccophora langsdorfii
Fucus vesiculosus
Dictyopteris divaricata
Vaucheria litorea
Gonyostomum semen
Vacuolaria virescens
Heterosigma akashiwo
Nannochloropsis oceanica
Nannochloropsis oculata
Nannochloropsis graculata
Nannochloropsis limnetica
Microchloropsis gaditana
Monodopsis sp. MarTras21
Vischeria sp. CAUP Q 202
Vischeria sp. ACOI3415
Characiopsis acuta
Pseudellipsoidion edaphicum
Paraeustigmatos columelliferus Mont 10/10-1w
Eustigmatophyceae sp. Bat 8/9-7w
Trachydiscus minutus
Eustigmatophyceae sp. Ndem 8/9T-3m6.8
Eustigmatophyceae sp. Chic 10/23 P-6w
Synura sphagnicola
Synura petersenii
Synura uvella
Mallomonas splendens
Neotessella volvocina
Hydrurus foetidus
"Ochromonas sp. CCMP1393"
Pedospumella sp. Jangsampo120217C5
"Spumella" sp. NIES-1846, AP019363
Spumella sp. Baeckdong012018B8
Poteriospumella lacustris Yongseonkyo072317C3
Chrysochromulina tobinii CCMP291
Chrysochromulina parva
Phaeocystis antarctica
Phaeocystis globosa
Emiliania huxleyi
Diacronema lutheri
Bacillariophyceae
Bolidophyceae
Pelagophyceae
Phaeophyceae
Xanthophyceae
Raphidophyceae
Eustigmatophyceae
Chrysophyceae
0.2
